# Trends in the treatment changes and medication persistence of chronic myeloid leukemia in Taiwan from 1997 to 2007: a longitudinal population database analysis

**DOI:** 10.1186/1472-6963-12-359

**Published:** 2012-10-16

**Authors:** Chao-Sung Chang, Yi-Hsin Yang, Chien-Ning Hsu, Min-Ting Lin

**Affiliations:** 1 Division of Hematology and Oncology, Department of Internal Medicine, Kaohsiung Medical University Hospital, Kaohsiung, Taiwan; 2 Graduate Institute of Healthcare Administration, Kaohsiung Medical University, Kaohsiung, Taiwan; 3 School of Pharmacy, Kaohsiung Medical University, Kaohsiung, Taiwan; 4 Cancer Center, Kaohsiung Medical University Hospital, Kaohsiung, Taiwan; 5 Department of Pharmacy, Kaohsiung Chang Gung Memorial Hospital and Chang Gung University College of Medicine, 123 Dabi Rd., Kaohsiung, 833, Kaohsiung, Taiwan; 6 Graduate Institute of Clinical Pharmacy, College of Pharmacy, Kaohsiung Medical University, Kaohsiung, Taiwan

**Keywords:** Chronic myeloid leukemia, Imatinib, Medication persistence, Utilization, Target therapy

## Abstract

**Background:**

Few studies have examined the longitudinal changes in the patterns, selection, and utilization of treatments for chronic myeloid leukemia (CML) in routine clinical practice since the introduction of imatinib. Therefore, we investigated the trends in CML therapy, including changes, patterns, and persistence to imatinib therapy among patients with newly diagnosed CML.

**Methods:**

We conducted a cross-sectional and longitudinal analysis of 11 years of claims data for patients with newly diagnosed CML included in the Taiwan National Health Insurance program. Pharmacy and diagnosis claims for newly diagnosed CML recorded between 1997 and 2007 year were extracted from the database. Annual overall use, new use of CML therapy, and persistence to imatinib therapy were estimated. The Anatomical Therapeutic Chemical codes for CML therapy [i.e., imatinib and conventional therapy: busulfan, hydroxyurea, interferon-α (IFNα), and cytarabine], and the process code for hematopoietic stem cell transplantation were used to categorize treatment patterns. Associations with patients characteristics were analyzed by multivariate logistic regression.

**Results:**

Overall, the proportion of patients with newly diagnosed CML to all patients with CML increased by approximately 4-fold between 1998 and 2007. There were steady increases in the proportions of all treated patients and those starting therapy from 2003 to 2007. Fewer comorbid conditions and lower severity of CML were associated with treatment initiation. Medication persistence varied according to treatment duration, as 38.7% patients continued imatinib for ≥ 18 months without interruption but only 7.7% continued imatinib for ≥ 5 years. Factors associated with persistence to imatinib therapy were removal of the need for prior authorization for imatinib, and prior use of hydroxyurea and IFNα, whereas having undergone hematopoietic stem cell transplantation led to reduced likelihood of persistence to imatinib therapy.

**Conclusion:**

Treatment decisions for patients with CML changed over time in routine clinical practice. Our findings suggest that clinicians are increasingly adopting the recommendations of international treatment guidelines for CML. However, persistence to imatinib therapy is still substantially below the recommended level based on current evidence for its efficacy. Our study also highlights the need to improve treatment persistence and effectiveness of imatinib over the long term.

## Background

Treatment guidelines established in 1998 recommend that chronic myeloid leukemia (CML) should be treated with conventional chemotherapy, interferon (IFN), or hematopoietic stem cell transplantation (HSCT) [1]. Although HSCT is considered a curative treatment for CML, patient eligibility, risk of early treatment-related mortality, and long-term debility because of chronic graft-versus-host disease have limited its application [2,3]. The treatment strategy for CML has changed substantially over the last decade following the introduction of specific targeted tyrosine kinase inhibitors (TKIs), with imatinib being one of the notable achievements in this class [4]. Imatinib is now widely used as first-line therapy for Philadelphia chromosome-positive CML in chronic phase (CP), accelerated phase (AP), and blast crisis (BC) based on the results of several clinical trials [5-7]. In addition, an 8-year update of the International Randomized Study of Interferon vs. STI571 (IRIS) trial demonstrated the durable efficacy of imatinib, as 55% (304/553) of the patients treated with imatinib were still on therapy at 8 years [8]. However, there is no information on medication interruption or duration of therapy beyond 8 years. It is also unclear whether the patterns of imatinib therapy in clinical trials were reflected by prescribers’ decision-making and the choice of therapy for CML in routine clinical practice.

Knowledge about the impact of imatinib on the types and patterns of treatments used to manage CML can help health planners to develop or refine their own reimbursement policies to optimize CML care. There are several advantages of using Taiwanese data to quantify treatment utilization and examine a drug’s potential public health impact. First, Taiwan provides universal health insurance coverage. The National Health Insurance (NHI) program offers a comprehensive benefits package, which includes reimbursement for prescription drugs, hospital care, and physician visits. Second, the system maintains a database that records claims and reimbursements for health services, and allows us to monitor the impact of adopting new drugs on resource utilization and quality of care. Finally, CML-related health services, including hospitalization, physician visits, and prescriptions, are fully reimbursed by the NHI program. Consequently, insured patients do not incur any out-of-pocket expenses for imatinib or CML therapy. In 2009, the Taiwan NHI program spent TW $12 billion (approximately US $4 million) on imatinib expenditure, which was ranked within the 10 highest amounts spent on individual drugs in that year. Therefore, it is important to examine the patterns of imatinib use in routine clinical practice from a payer perspective. Consequently, the objectives of this study were to determine the longitudinal trends in CML therapy, explore the patterns of use and medication persistence to imatinib, and identify possible demographic factors influencing its use in patients with newly diagnosed CML enrolled in Taiwan’s NHI program.

## Methods

### Study design

We conducted a cross-sectional study of patients with newly diagnosed CML enrolled in the Taiwan NHI program to estimate the proportion of patients treated for CML over 10 consecutive years. CML therapies included HSCT and regimens commonly used to treat CML-CP, including imatinib, busulfan, hydroxyurea, and IFNα with or without cytarabine (Ara-C). To analyze persistence to imatinib therapy, we identify treatment-naïve patients and tracked them from the date of starting treatment (index date) until the any of the following: death in hospital, transfer to hospice services, HSCT, or the end of the study period (December 2007), whichever came first.

### Data source

All data were retrieved from the National Health Insurance Research Database (NHIRD) provided by the Bureau of National Health Insurance (BNHI), Department of Health, Taiwan. The National Health Research Institutes (NHRI) maintains the health claims database, including registration files and original inpatient, outpatient, and pharmacy claims within the NHI program. These data files are de-identified by scrambling the identification codes of both patients and medical facilities. The NHIRD adheres to the Computer-Processed Personal Data Protection Law and related regulations of the BNHI and the NHRI [9].

Health insurance claims data used in this study represented actual payments made to the NHI program between January 1, 1997, and December 31, 2007. Because the NHI provides compulsory enrollment and has a high coverage rate (99.3% in 2007), all census regions were considered to be well represented over time in the database [10].

The study was approved by the Institutional Review Board at Chung-Ho Memorial Hospital, Kaohsiung Medical University.

### Study population

We first identified all individuals with at least one claim associated with a diagnosis of CML-CP (International Classification of Disease Version 9 Clinical Modification [ICD9-CM] code 205.1x) for inpatient or outpatient claims submitted between 1997 and 2007. Newly diagnosed CML was defined as (1) previously undiagnosed CML-CP; (2) previously undiagnosed CML-AP/BC, or history of remission; (3) previously undiagnosed acute leukemia; or (4) no prior CML therapy, including HSCT, imatinib, busulfan, hydroxyurea, and IFNα with or without Ara-C. We do not report CML therapy for 1997 because medical and treatment history prior to January 1997 are not available in this database. The high severity category in the Medstat Disease Staging Clinical Criteria Version 5.24 was used as a proxy for patients with CML-AP/BC [11].

### Study measures

#### Utilization of CML therapy

The utilization of CML therapy was determined from pharmacy claims using the Anatomical of Therapeutic Chemical codes for imatinib, conventional regimens (i.e., busulfan, hydroxyurea, IFNα, and Ara-C), and the procedure codes for HSCT. Drug utilization during hospitalization and in outpatient settings during each calendar year was retrieved for patients with newly diagnosed CML-CP.

Annual CML therapy utilization was calculated as the proportion of patients with newly diagnosed CML with at least one claim for CML therapy or HSCT, including those who were ‘ever treated’ during each calendar year. To determine trends in treatment patterns, the proportions of patients who started treatment for CML were categorized into three groups: HSCT (exclusively to imatinib), imatinib (exclusively to HSCT), and CML conventional therapy (busulfan, hydroxyurea, IFNα, and Ara-C, alone or in combination).

Accordingly, the annual proportion of patients starting CML therapy was determined for patients with newly diagnosed CML with a first claim for a CML regimen or HSCT in each calendar year. We next identify demographic factors associated with the longitudinal trends in treatment utilization and retrieved the following factors from the database: patient characteristics at baseline, including age at diagnosis, sex, year of CML diagnosis classified as before or after the introduction of imatinib in Taiwan (before 2004 vs 2004 or later), CML severity (i.e., low, moderate, or high, based on the Medstat algorithm) at 4 months after CML diagnosis, and Charlson Comorbidity score (CCS) for comorbidities 1 year before CML diagnosis. These factors were determined for newly diagnosed patients before the approval of imatinib (1998), at removal of the prior authorization request for imatinib (2004), and after full access to imatinib use (2007). We also determined the effect of insurance coverage on the start of imatinib therapy [12] using multivariate logistic regression in which we determined the likelihood of receiving imatinib according to patient characteristics at baseline in 1998, 2004, and 2007.

#### Patterns of imatinib prescription

Patterns of imatinib prescription were defined according to the mean starting dose, time of starting imatinib, time of discontinuation, and prior use of CML therapy. The mean starting dose (mg/day) was calculated as the number of pills taken (converted into mg; ×100 mg/tablet) divided by the length of time (days) between the first two consecutive claims. Only patients starting imatinib with at least two claims for imatinib therapy were analyzed; the starting daily dose was not calculated in patients with a single claim throughout the study period. The time between CML diagnosis and starting imatinib therapy was calculated in months. Discontinuation was defined as the last day of a 30-day supply of imatinib in the last recorded claim. Treatment duration was calculated as the time from starting imatinib therapy (index date) to the date of discontinuation. Prescriptions extending beyond the end of the follow-up were truncated at the last day of the follow-up period.

#### Medication persistence

Medication persistence was measured as the duration of initial imatinib therapy without interruption [13]. Medication interruption was defined as a gap of ≥ 60 days between imatinib claims with the assumption that patients could refill their imatinib prescription within 30 days from the run-out date of the previous prescription refill without compromising treatment outcomes. The duration of initial imatinib therapy without interruption was calculated the time from the index date to the run-out date of the last claim (the date of the last claim plus 30 days) before an interruption. Previously with newly diagnosed CML-CP who failed imatinib therapy were identified as those fulfilling one or more of the following criteria: no complete hematologic response after 3 months of initial therapy, no major cytogenetic response after 6 months of therapy, no complete cytogenetic response after 18 months of therapy, hematologic or cytogenetic relapse at any time, progression to CML-AP or CML-BC at any time, treatment discontinuation, or HSCT [14,15]. To provide better insight into medication persistence within 18 months of starting therapy in real world settings, the duration of initial imatinib therapy without interruption was classified as < 18 months (non-persistent) and ≥ 18 months (persistent). Multivariate logistic regression was used to estimate the likelihood of treatment persistence according to patient characteristics at baseline, prior treatment, time of starting imatinib therapy, mean starting dose of imatinib, treatment duration, and HSCT during the follow-up period. Data management and analyses were conducted using SAS Version 9.1 (Cary, NC).

## Results

### Patient characteristics

Our analysis sample consisted of 2,672 patients, after excluding patients with a history of remission, advanced CML, acute leukemia, or CML therapy before the diagnosis of CML-CP. The estimated annual incidence of CML in the study population was 1.2 cases per 100,000 persons (standard deviation = 0.2) between 1998 and 2007, and was steady at 1.1 cases per 100,000 persons (SD = 0.1) between 2003 and 2007. The mean age at diagnosis was 55.7 ± 20.4 years; 1,264 patients were > 60 years old (47.3%). Overall, there were more men than women with newly diagnosed CML (60.8% and 39.2%, respectively). Based on the Medstat algorithm, the severity of CML was graded as high, moderate, and low in 12.4%, 72.2%, and 2.1%, respectively; CML severity was missing in 13.3%. Over half of the patients (53.8%) had at least one comorbidity (CCS ≥ 1).

### CML therapy

**Figure 1:**
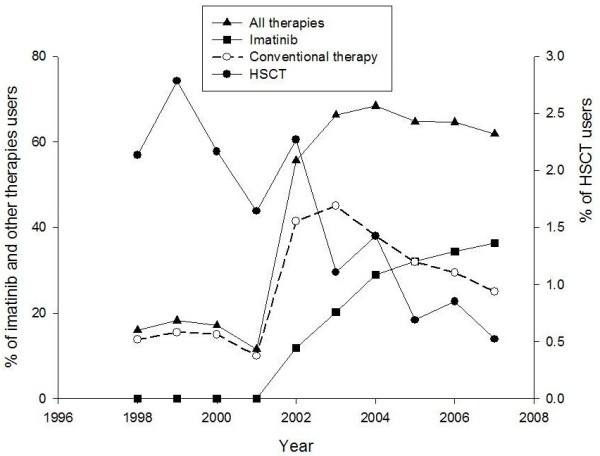
CML therapy overall use in patients with newly diagnosed CML per calendar year (1998-2007).

Figure 1 presents the longitudinal changes in CML therapy. The proportion of patients undergoing HSCT decreased gradually over the 10-year period from 3% in 1999 to 1% in 2007. The proportion of patients treated with imatinib increased from 12% in 2002 to 36% in 2007. The proportion of patients treated with conventional CML therapies (i.e., hydroxyurea, busulfan, IFNα, and Ara-C alone or in combination) was determined for three different periods, 1998–2001 (the rate decreased slightly from 14% to 10%), 2002–2003 (the rate increased slightly from 41% to 45%), and 2004–2007 (the rate decreased significantly from 38% to 25%). The most commonly used conventional therapy was hydroxyurea followed by IFNα with/without Ara-C and busulfan.

**Figure 2:**
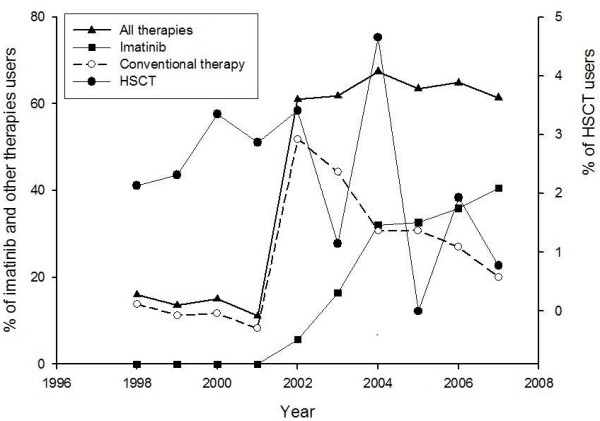
CML therapy new use in patients with newly diagnosed CML per calendar year (1998-2007).

Figure 2 shows the trends in initial treatment type for CML. The proportion of patients receiving new CML therapy increased approximately 4-fold over the 10-year period (from 42 of 281 in 1998 to 156 of 258 in 2007). Imatinib became a dominate treatment when the need for prior authorization was stopped in 2004. The use of conventional regimens tended to increase between 2002 and 2003, but then declined following broad access to imatinib.

As indicated in Table 1, we found temporal changes in the associations between patient demographic factors and the start of treatment for CML. Patients > 40 years old were less likely than younger patients to start CML therapy before (i.e., in 1998) and after the introduction of imatinib (i.e., 2004 and 2007). There was no difference between males and females in the likelihood of receiving therapy. Patients with a comorbidity were more likely to receive therapy than patients without a comorbidity in 1998 [odds ratio (OR), 1.2; 95% confidence interval (CI), 0.6–2.4]. However, following the introduction of imatinib, there was a trend towards a reduced likelihood of starting treatment among patients with a comorbidity in 2004 (OR, 0.6; 95% CI, 0.4–1.2) and 2007 (OR, 0.4; 95% CI, 0.2–0.6). Similarly, the presence of advanced CML decreased the likelihood starting therapy in the same year of diagnosis in 1998 (OR, 4.3; 95% CI, 2.0–9.3), 2004 (OR, 2.1; 95% CI, 0.8–5.4) and 2007 (OR, 2.3; 95% CI, 1.1–5.1), although the level of significance was not maintained throughout this period.

**Table 1:** Associations between receiving Chronic Myeloid Leukemia (CML) treatment initiation and patient characteristics

**Characteristics**	**1998**^**a**^				**2004**				**2007**			
	**n (%)**	**χ2**	**OR**	***p***	**n (%)**	**χ2**	**OR**	***p***	**n (%)**	**χ2**	**OR**	***p***
		**(p-value)**	**(95%CI)**^**b**^			**(p-value)**	**(95%CI)**			**(p-value)**	**(95%CI)**	
**Age at diagnosis, years**												
**≥40**	228 (81.1)	9.16 (<0.01)	0.4 (0.2-0.8)	<0.01	160 (74.4)	6.30 (0.01)	0.5 (0.2-0.9)	0.03	186 (72.1)	16.86 (<.0001)	0.4 (0.2-0.7)	<0.01
**<40**	53 (18.9)		1 (referent)		55 (25.6)		1 (referent)		72 (27.9)		1 (referent)	
**Gender**												
**Male**	166 (59.1)	0.00 (0.95)	0.8 (0.4-1.7)	0.60	126 (58.6)	1.25 (0.26)	0.7 (0.4-1.3)	0.26	151 (58.5)	0.49 (0.49)	1.4 (0.8-2.3)	0.29
**Female**	115 (40.9)		1 (referent)		89 (41.4)		1 (referent)		107 (41.5)		1 (referent)	
**Charlson comorbidity score**												
**≥1**	101 (35.9)	0.00 (0.97)	1.2 (0.6-2.4)	0.71	118 (54.9)	3.71 (0.05)	0.6 (0.4-1.2)	0.13	140 (54.3)	20.36 (<.0001)	0.4 (0.2-0.6)	<0.001
**=0**	180 (64.1)		1 (referent)		97 (45.1)		1 (referent)		118 (45.7)		1 (referent)	
**CML severity**												
**high severity**	45 (16.0)	17.90 (<.0001)	4.3 (2.0-9.3)	<0.001	28 (13.0)	1.64 (0.20)	2.1 (0.8-5.4)	0.12	40 (15.5)	2.87 (0.09)	2.3 (1.1-5.1)	0.04
**less severity**^**c**^	236 (84.0)		1 (referent)		187 (87.0)		1 (referent)		218 (84.5)		1 (referent)	

a. 1998 (beginning of study, n = 281); 2004 (end of prior authorization request for imatinib use, n = 215); 2007 (end of study, n = 258).

b. OR = odds ratio, each OR of the variable was adjusted by the other factors in the logistic regression model.

c. Less severity, including moderate, low severity or missing relevant severity information (unknown).

### Patterns and persistence of imatinib therapy

The mean age of patients starting imatinib therapy (n = 782) was 47.8 years. Approximately 60% of these recipients were male, 49.4% were diagnosed with CML in 2002 or later, and 43% had at least one comorbidity. The severity of CML was high in 12.4% of patients. Overall, 72% of the patients had been treated with hydroxyurea, 18% with IFNα/Ara-C, and 2.2% with busulfan before starting imatinib. The median time from diagnosis to starting imatinib therapy was 3.1 months (mean 14.6 ± 19.5 months). In most patients (73%), the starting dose was 300–400 mg/day, while 16.3% started at a dose ≤ 300 mg/day, and 10.7% started at a dose ≥ 400 mg/day. The mean ± SD duration of imatinib treatment was 26.1 ± 19.9 months, and ranged from 1 to 69.8 months. With over 5 years of follow-up data (range, 0.2–69 months), imatinib therapy was discontinued in 269 (34.4%) patients. Reasons for discontinuation included HSCT (3%), death/transfer to hospice services (7.7%), disenrollment and/or end of follow-up (19.4%), and unknown reasons (4.6%). Approximately one-third of patients (33.4%) received imatinib for < 1 year, 20% for 1–2 years, 13.4% for 2–3 years, 17.1% for 3–4 years, 8.6% for 4–5 years, and 7.7% for > 5 years.

Table 2 shows the baseline characteristics and patterns of imatinib use for persisting and non-persisting patients. Overall, 38.7% (n = 303) of patients were defined as persistent in their initial treatment period. Patients diagnosed with CML after the withdrawal of imatinib prior authorization (2004 or later) (OR, 4.1; 95% CI, 2.2–7.9), previously treated with hydroxyurea (OR, 1.8; 95%CI, 1.1–2.8) and IFNα or Ara-C (OR, 1.9; 95% CI, 1.1–3.3), and longer duration of imatinib therapy (OR,1.1; 95% CI, 1.09–1.13) were significantly associated with an increased likelihood of persistence to imatinib therapy. Patients undergoing late HSCT were less likely to persist with initial imatinib therapy (OR, 0.1; 95% CI, 0.02–0.50).

**Table 2:** Factors associated with the persistence of imatinib initial therapy

**Characteristics**	**Non-persistent**^**a**^	**Persistent**	**χ2**	**OR for being persistent (95% CI)**	**p value**
	**(n = 479)**	**(n = 303)**	**(p-value)**		
Age at diagnosis, y, n (%)			2.08 (0.15)		
≥40,	308 (64.3)	210 (69.3)		1.4 (0.9 to 2.1)	0.15
<40	171 (35.7)	93 (30.7)		1 (referent)	
Gender, n (%)			0.44 (0.51)		
Male	281 (58.7)	185 (61.1)		1.4 (0.9 to 2.1)	0.10
Female	198 (41.3)	118 (38.9)		1 (referent)	
Charlson comorbidity score, n (%)			1.39 (0.24)		
>0	215 (44.9)	123 (40.6)		0.8 (0.5 to 1.1)	0.16
=0	264 (55.1)	180 (59.4)		1 (referent)	
CML diagnosis, n (%)			11.97(<0.001)		
≥2004	260 (54.3)	126 (41.6)		4.1 (2.2 to 7.9)	<.0001
<2004	219 (45.7)	177 (48.4)		1 (referent)	
CML severity, n (%)			7.60 (0.06)		
Unknown	28 (5.9)	24 (7.9)		0.6 (0.2 to 1.6)	0.20
Low	2 (0.4)	2 (0.7)		1.4 (0.1 to 19.0)	0.77
Moderate	378 (78.9)	251 (82.8)		1.5 (0.8 to 3.0)	0.30
High	71 (14.8)	26 (8.6)		1 (referent)	
Prior treatment, n (%)					
Hydroxyurea	342 (71.4)	225 (74.3)	0.76 (0.38)	1.8 (1.1 to 2.8)	0.02
Busulfan	8 (1.7)	9 (3.0)	1.48 (0.22)	0.5 (0.1 to 2.0)	0.34
IFNα/Ara-C	72 (15.0)	75 (24.8)	11.49(<0.001)	1.9 (1.1 to 3.3)	0.02
Mean time to imatinib initiation, month (SD)	14.1 (19.6)	15.4 (19.4)	0.89 (0.37)^d^	1.0 (0.99 to 1.02)	0.42
Mean starting daily dose, mg/day (SD)^b^	362 (141)	375 (108)	1.35 (0.18) ^d^	1.1 (0.97 to 1.34)	0.11
Mean treatment duration, months (SD)	16.5 (16.8)	41.2 (14.2)	21.94 (<.0001) ^d^	1.1 (1.09 to 1.13)	<.0001
HSCT post-index date, n (%)	35 (7.3)	2 (0.7)	18.19(<.0001)	0.1 (0.02 to 0.5)	0.01

a. Patients were considered persistent if they were taking imatinib without interruption (exceeding a 60-day permissible gap) for at least 18 months since the start of therapy.

b. Excluding patients with a single imatinib claim (n = 19).

c. OR = odds ratio, each OR of the variable was adjusted by the other factors in the logistic regression model.

Abbreviations: *CML*, chronic myeloid leukemia; I*FN*α, interferon-α; *Ara-C*, cytarabine; *HSCT*, hematopoietic stem cell transplantation.

d. *t* test for numerical outcomes.

## Discussion

The results of this study provide important insight into the status of CML therapy in Taiwan. First, the results are consistent with the current clinical guidelines recommending imatinib as first-line therapy for CML of any stage. The use of imatinib increased rapidly between 2002 and 2004, with an increase of over 5-fold during this time, corresponding to the introduction of imatinib in Taiwan. We also found that the proportion of patients with very severe CML starting imatinib decreased significantly from 38.1% to 18.6% between 1998 and 2007, which suggests that imatinib was mostly started in CML-CP. This may be due to the results of the IRIS trials, which showed that the complete cytogenetic response rate was lower in patients with CML in advanced phases as compared with earlier phases of CML [6,7]. Future studies should examine the appropriateness of very frequent imatinib use and whether starting treatment in earlier stages provides better outcomes.

Second, the impact of TKIs on the trends in conventional CML regimens is quite pronounced. Consistent with the results of the European Group for Blood and Marrow Transplantation activity survey [2], the number of patients treated with HSCT gradually declined in our study (from 10 cases in 2004 to 2 cases in 2007). Although busulfan was also used before the introduction of TKIs, its utilization remained low after the introduction of imatinib. On the other hand, we found temporal relationship between the utilization of imatinib and either hydroxyurea or IFNα between 2002 and 2003. Although hydroxyurea does not appear to cure or modulate the progression of disease, its concomitant use with imatinib may help to control early symptoms, particularly increases in the white blood cell count. Although IFNα is superior to hydroxyurea and busulfan in terms of clinical outcomes [16], it was less widely used, and the proportion of patients treated with IFNα decreased markedly between 2003 and 2004. One explanation for this is that IFNα is associated with considerable adverse effects. It must be noted that some patients treated with imatinib were also concurrently treated with other conventional regimens in any year. The differences and trends in utilization observed here suggest that the choice of treatment for CML was probably influenced by treatment responses (e.g. failure, intolerant drug adverse effect), clinical evidence and the need for prior authorization to use imatinib in accordance with the NHI program at the time. The effects of using these conventional drugs in combination with imatinib on long term clinical outcomes remain uncertain.

Interestingly, the time from starting to discontinuing imatinib therapy differed substantially between clinical trials and routine clinical practice. Because the definition of persistence in our study was linked to the length of continued treatment, we found that a large proportion of newly diagnosed patients were only briefly treated with imatinib. Unlike the IRIS trial, we found that approximately one-third (33.4%) of patients who started imatinib therapy discontinued or interrupted treatment within 1 year, and < 10% of patients continued imatinib therapy for > 5 years, and 55.5% of the newly diagnosed patients did not receive imatinib within 6 months of diagnosis. Although some of the patients continued imatinib for > 18 months and some for the entire treatment period, drug interruptions caused by non-adherence, for example, can result in failure to achieve an adequate molecular response, and may compromise event-free survival [17,18].

Third, previous studies have shown that the adherence rate to imatinib therapy in clinical settings are less than optimal. Some studies use the medication possession rate (MPR), which is defined as the total number of days’ supply of imatinib divided by the duration of follow-up to assess medication adherence. Neoens et al. conducted a multicenter prospective study of patients treated with imatinib for ≥ 30 days over a 90-day period [19]. They found that only 14.2% of the patients fully adhered to the prescribed imatinib. Marin et al. reported that over a 1-year follow-up, 74.6% of patients had an MPR > 90% determined using a microelectronic pill counting system in their single-center study [20]. Wu et al. retrospectively assessed MPR in patients with newly diagnosed CML enrolling in a managed care organization [21]. In that study, 59.1% of patients newly treated with imatinib had an MPR ≥ 85% during the 1-year follow-up. Darkow et al. also studied managed-care recipients diagnosed with CML, and found that 31% of the patients had a treatment interruption and 50% had an MPR > 95% over 1 year of follow-up [22]. Although the cutoff time used to define non-adherence and the results of other studies are limited to a relatively short duration of therapy [21,22], poor compliance to imatinib therapy may actually increase healthcare costs and prevent optimal clinical outcomes.

Persistence adds the dimension of time to the analysis and represents the time from the initial filling of the imatinib prescription until the patient discontinues (or interrupts) prescription refills [13]. We noted an increased likelihood of non-persistence to imatinib within 1 year of CML diagnosis in patients diagnosed earlier than 2002. This may be due to the need for prior authorization for imatinib according to the NHI program, which was necessary between January 2002 and December 2003. The requirement for prior authorization probably created an unintended barrier that affected the physician’s decisions and likelihood of continuing mediation during the initial period of treatment. We found that better medication persistence at the beginning of imatinib therapy (≧ 18 months) was associated with a longer treatment duration during the follow-up and prior therapies with hydroxyurea and IFNα (or Ara-C). There are several possible reasons for this finding. For example, the patients are more likely to experience intolerable adverse effects or poor efficacy, or the patient discontinue for symptomatic relief. However, the severity of CML at diagnosis and the time of starting treatment after diagnosis were not significantly associated with persistence in our study. Usually, patients with severe or complex disease are more likely to start therapy sooner after diagnosis and are more likely to be adherent (for example, prior therapies with hydroxyurea, IFNα or Ara-C) than those with asymptomatic disease. However, it is possible that patients with advanced disease are more likely to suffer severe hematologic responses that require treatment discontinuation, and hence incur additional health costs [21,22]. These hypotheses should be investigated in this population.

Imatinib provided a major advance in the treatment of CML. Current guidelines recommend imatinib as first-line therapy for CML-CP, and should also be considered as a treatment option for CML-AP, CML-BC or CML-CP after failure of IFNα therapy [14,15]. However, a substantial proportion of patients may need clinical support to persist with therapy and hence achieve the optimal benefits of imatinib. As described by other investigators, non-adherence to therapy may be represent an interaction between patient, healthcare professional, economic, and healthcare system factors [19,23]. Persistence to initial therapy should be an achievable target in routine clinical practice for managing CML in all stages. As imatinib is fully reimbursed by the NHI program, interventions are needed to enhance treatment persistence. Such interventions should encourage physicians to regularly monitor and continue to follow-up patients indicated for and starting imatinib therapy. Unlike many parenteral anticancer medicines, patients taking oral imatinib do so on their own. Therefore, we should endeavor to identify patients at high risk of non-persistence. We may be able to provide them and their caregivers with appropriate education to better understand the clinical benefits of persistence and adherence to therapy [19], even though their disease may be improving during treatment. Supportive activities and individual interventions aimed at breaking down the barrier to adherence may reinforce medication persistence and adherence.

A limitation to our study is the lack of laboratory data that can be used to assess the severity of CML in newly diagnosed patients. To overcome this limitation, we used the Medstat disease staging algorithm, which is widely applied to measure the different phases of disease and analyze resource utilization, reimbursement, and quality of care. Compared with neighboring Asian countries [12], ≤ 20% of the new CML diagnoses were CML-AP (mean, 11%; range, 0–30%) and ≤ 15% were CML-BC (mean, 5%; range, 0–20%). Thus, our data are generalizable to the Asia-Pacific region. Unfortunately, because of the aggregated nature of hospitalization and outpatient visit claims, the duration of drug prescription and daily dosing frequency in medical records cannot be accurately assessed using claims data alone. We tried to overcome this limitation by including the mean daily dose in patients with at least two claims for imatinib. The mean starting daily dose for some non-persistent patients was much lower than the recommended dose of 400 mg. This may be because these patients refilled the prescription for imatinib after a long gap (e.g., > 60 days) and tended to have a short duration of treatment (< 1 year). Therefore, the starting dose in these patients is likely to be underestimated. Consequently, the association between the mean starting dose and medication persistence may be biased towards a high starting dose. Another limitation of using claims data is that we cannot confirm whether the drug was actually taken or taken as prescribed. Finally, patient-related factors (e.g., cognitive ability, lack of family support, and limited understanding of the need for long-term therapy) may influence adherence, but could not be accounted for in this study.

## Conclusions

This population-based observational study revealed that the use of imatinib to treat newly diagnosed CML has increased over the last decade, principally because of increased prescription in patients with fewer comorbidities and less-severe CML. Although imatinib is widely available, does not need prior authorization anymore, and is fully reimbursed, a large number of patients starting imatinib discontinued therapy within a short time or did not persist with therapy for the critical initial therapeutic period. To achieve optimal therapeutic outcomes, it is essential that the patient persists with therapy, which relies on ongoing monitoring, encouragement, education, and regular follow-up. From the healthcare system perspective, further studies are needed to evaluate methods to improve persistence as well as outcomes, both effectiveness and risks associated with long-term imatinib therapy.

## Competing interests

The authors declare that they have no competing interests.

## Authors' contributions

CSC helped to conceive of the study and revised the manuscript; YSY obtained funding and supplied the database; MTL performed data analyses; CNH helped to conceive of and design the study, and wrote the manuscript. All authors approved the final submitted manuscript.

## Pre-publication history

The pre-publication history for this paper can be accessed here:

http://www.biomedcentral.com/1472-6963/12/359/prepub
